# GWAS of CRP response to statins further supports the role of APOE in statin response: A GIST consortium study

**DOI:** 10.1016/j.phrs.2024.107575

**Published:** 2025-01-09

**Authors:** Emma F. Magavern, Harshal Deshmukh, Geraldine Asselin, Elizabeth Theusch, Stella Trompet, Xiaohui Li, Raymond Noordam, Y.-D. Ida Chen, Teresa E. Seeman, Kent D. Taylor, Wendy S. Post, Jean-Claude Tardif, Dirk S. Paul, Emelia J. Benjamin, Nancy L. Heard-Costa, Ramachandran S. Vasan, Jerome I. Rotter, Ronald M. Krauss, J.Wouter Jukema, Paul M. Ridker, Patricia B. Munroe, Mark J. Caulfield, Daniel I. Chasman, Marie-Pierre Dubé, Graham A. Hitman, Helen R. Warren

**Affiliations:** a Centre of Clinical Pharmacology & Precision Medicine, William Harvey Research Institute, Queen Mary University of London, London, UK; b NIHR Barts Biomedical Research Centre, Queen Mary University of London, London, UK; c Mackay Base Hospital, Queensland Health, Queensland, Australia; d Faculty of Medicine, Université de Montréal, and the Montreal Heart Institute, Montreal, Canada; e Department of Pediatrics, University of California San Francisco, Oakland, CA, United States; f Department of Cardiology, Leiden University Medical Center, Leiden, the Netherlands; g Department of Internal Medicine, Section of Gerontology and Geriatrics, Leiden University Medical Center, Leiden, the Netherlands; h Institute for Translational Genomics and Population Sciences, Department of Pediatrics and The Lundquist Institute at Harbor-UCLA Medical Center, Torrance, CA, USA; i Division of Geriatrics, Dept of Medicine, University of California Los Angeles, Los Angeles, CA, USA; j Division of Cardiology, Department of Medicine, Johns Hopkins University, Baltimore, MD, USA; k Centre for Genomics Research, Discovery Sciences, BioPharmaceuticals R&D, AstraZeneca, Cambridge, UK; l Precision Medicine and Biosamples, Oncology R&D, AstraZeneca, Cambridge, UK; m Boston University Chobanian & Avedisian School of Medicine and School of Public Health, NHLBI and Boston University’s Framingham Heart Study, Framingham, MA, United States; n Division of Preventive Medicine, Brigham and Women’s Hospital, and Harvard Medical School, Boston, MA, United States; o Centre of Genomic Medicine and Child Health, Blizard Institute, Queen Mary University of London, London, UK

**Keywords:** Statins, C-Reactive Protein, Pharmacogenetics, GWAS, Treatment Response, Pharmacology

## Abstract

Statins are first-line treatments in the primary and secondary prevention of cardiovascular disease. Clinical studies show statins act independently of lipid-lowering mechanisms to decrease C-reactive protein (CRP), an inflammation marker. We aim to elucidate genetic loci associated with CRP statin response.

CRP statin response is the change in log-CRP between off-treatment and on-treatment measurements. Cohort-level Genome-Wide Association Studies (GWAS) of CRP response were performed using 1000 Genomes imputed data, testing ~10 million common genetic variants. GWAS meta-analysis combined results from seven cohorts and clinical trials totalling 14,070 statin-treated individuals of European ancestry within the GIST consortium. Secondary analyses included statin-by-placebo interaction analyses, and lookups in African ancestry cohorts.

Our GWAS identified two genome-wide significant (P < 5e-8) loci: *APOE* and *HNF1A* for CRP statin response corrected for baseline CRP. The missense lead variant rs429358 at *APOE*, contributing to the *APOE*-E4 haplotype, is a risk locus for dyslipidaemia, Alzheimer’s and coronary artery disease (CAD). The *HNF1A* locus is associated with diabetes, cholesterol levels, and CAD. Both loci are also associated with baseline CRP levels, and neither locus achieved a significant (P < 0.05) result from the statin v. placebo interaction meta-analysis using randomized clinical trial data. However, the interaction result (P-int=0.09) for *APOE* was suggestive and possibly underpowered.

The *APOE*-E4 signal may therefore be associated with both CRP and LDL-cholesterol statin response. Combined with suggestions in the literature that *APOE* also leads to differential statin benefit in Alzheimer’s, the *APOE* locus warrants further investigation for potential genetic effects on healthcare with statin treatment.

## 1. Introduction

Statin medications, which were developed to lower low-density lipoprotein (LDL)-cholesterol and reduce risk of cardiovascular events, are first line treatments in the primary and secondary prevention of cardiovascular disease [1]. Prior clinical studies have shown that statins act independently of lipid lowering mechanisms to decrease C-reactive protein (CRP), a marker of inflammation [2–4]. However, the mechanism of action by which statins achieve this well replicated anti-inflammatory effect has remained largely a mystery in the more than two decades since its discovery.

There is known to be variability in response to statins [5]. Genomic variation is one factor contributing to this inter-individual variability [6]. The field of genomic effect on medication response is referred to as pharmacogenomics. The Genomic Investigation of Statin Therapy (GIST) consortium is comprised of a group of investigators utilising clinical trials and observational studies to analyse the genome-wide genetic effects on statin response, i.e. pharmacogenomics, across multiple phenotypes, such as LDL-cholesterol, high density lipoprotein (HDL)-cholesterol, myocardial infarction risk, and type 2 diabetes [6–9].

The only prior study to investigate the genomic underpinning of CRP response to statins is an analysis of the JUPITER trial [10]. The investigators analysed approximately 7000 participants of European ancestry who were randomly allocated to either placebo or 20 mg per day rosuvastatin [10]. In addition to a Genome-Wide Association Study (GWAS) for CRP statin response, the study focused on analyses of seven candidate genes known for LDL-statin response, and 20 candidate genes known for CRP-association, to test whether LDL-statin response genes or CRP-associated genes also influence CRP response to statins. However, very few of the results survived multiple testing correction. Therefore, the investigators concluded that the genomic loci associated with LDL-cholesterol response were not associated with CRP response to rosuvastatin ^10^. However, detection of genetic effects on CRP response that were smaller than those detected for LDL response may have been limited by power in the agnostic genome-wide mode of analysis.

The objective of this study was therefore to conduct the first ever large-scale pharmacogenetic GWAS meta-analysis for CRP response to statins, within the GIST Consortium, to elucidate genetic loci associated with CRP response to statins, and to gain mechanistic insight into the reduction of inflammation demonstrated in individuals taking statins.

## 2. Methods

### 2.1. Cohort level genome wide association studies (GWAS)

The CRP response phenotype was defined as the change in log CRP between baseline off-treatment and on-treatment measurement after at least four weeks of statin therapy (log(CRP follow-up/CRP baseline)). Each of the seven participating studies within the GIST consortium (CARDS, FHS, JUPITER, MESA, PARC, PROSPER, MHI-AstraZeneca cohort) performed the cohort level GWAS analysis of cohort level CRP response to statin therapy. The analysis was adjusted for baseline CRP (log transformed) as well as age, sex, BMI, and the first 10 genetic principal components by inclusion as covariates. The use of baseline CRP as a covariate mitigates prominence of loci which were only associated with baseline CRP as opposed to pharmacogenomic response of CRP to statins. Genetic data were imputed to 1000 Genomes or TOPMed (MHI-AstraZeneca cohort) imputation reference panels, testing ~10 million single nucleotide polymorphisms (SNPs) of common frequency. As most cohorts with available data were predominantly European ancestry, any individuals of non-European ancestry, and those missing phenotype or covariate information were excluded from the primary discovery analysis. (A subsequent secondary analysis in individuals of African ancestry is later performed, for comparison to the European ancestry discovery results.) An additive genetic model was assumed to test for association by regressing the response variable onto the total dose of the coded allele (e.g. AA=0, AG=1, GG=2 if G is the coded allele) at each SNP, assuming a normal linear regression model. For imputed SNPs, regression was performed onto expected allele dosage. The MHI-AstraZeneca cohort included eight randomized clinical trial studies (AURORA, CORONA, GRAVITY, LUNAR, METEOR, PLANETI, PLANETII, SATURN), with different inclusion criteria and dosages of statin, which were analyzed as a mega-analysis with additional adjustment for dose equivalents at cohort level prior to being included in the meta-analysis as a single study (see supplementary materials) [6]. The other study cohorts, which were comprised of single rather than multiple studies, did not adjust for different statins/doses with dose equivalents (Supplementary Table 1D)*”*.

### 2.2. GWAS quality control and meta-analysis

The EasyQC package in R statistical software was used to conduct central quality control (QC), to harmonize and standardize the cohort level data, restricted to imputation quality with INFO score > 0.4 and minor allele frequency (MAF) ≥ 2 % [11]. We then performed inverse-variance weighted fixed-effects meta-analysis using METAL software to combine the cohort level GWAS data results from the seven different studies of the GIST consortium [12], and subsequently conducted further QC of the meta-analysed data, restricting the output to variants which were present in ≥ 2 studies, and with a heterogeneity p-value< 1 × 10^−4^.

### 2.3. Significance reporting

SNPs were declared as significant from the primary GWAS meta-analysis if they reached genome-wide significance level (P < 5 x10^8^). Loci were defined according to 1 Mb loci regions, centred + /−500kb around the lead SNP.

### 2.4. Evaluation of results

Quantile-Quantile (QQ) plots and Manhattan plots of the overall GWAS meta-analysis results were generated using the qqman package in R. The heterogeneity of the meta-analysis results was checked. Locus-Zoom was used to visualize the top loci [13]. Forest plots were generated using the rmeta package in R to check the QC and robustness of the top loci.

PhenoScanner, a curated database of GWAS results, was used to investigate whether any of the signals reaching genome-wide significance are known to be associated with any other disease traits, by searching for GWAS trait associations reaching genome-wide significance for the lead SNP plus any nearby proxies (EUR-LD r^2^>0.7) [14]. PhenoScanner was also used to identify any significant (P < 5 ×10^−8^) eQTLs associations for the two signals reaching significance from this CRP statin response GWAS meta-analysis, across all available tissue types [14].

### 2.5. Heritability analysis

LD Score regression (LDSC) software (v1.0.1) was used to estimate the heritability of CRP response from the GWAS meta-analysis summary level statistics [15]. LDSC was applied to the GWAS meta-analysis summary statistics that had been filtered to include only variants present in the HapMap project (~1.1 million variants), along with precomputed LD scores using the 1000 G European ancestry reference panel provided by LDSC.

### 2.6. Conditional analysis

Genome-wide conditional analysis was performed to check for any independent secondary signals by running a “cojo” analysis using GCTA (Genomic Complex Traits Analysis) software, which iteratively implements joint conditional and stepwise multivariate analyses [16,17]. The primary GWAS meta-analysis summary statistics were used as the input file, and the publicly available 1000 Genomes reference dataset of n = 503 individuals of European ancestry (CEU) was used as the raw cohort-level genetic dataset for the estimation of LD. A genome-wide significance level (P < 5 ×10^−8^) was used to report any secondary signals that remained in the final output of jointly independently associated variants.

### 2.7. Interaction analysis: effect of statin vs placebo allocation on CRP response

To confirm that the effects on statins on CRP levels were modified by the two loci which were significant at genome-wide level from the meta-analysis, we also performed an interaction analysis using data from the randomized clinical trials (CARDS, PROSPER, JUPITER, and three of the MHI-AstraZeneca trials: AURORA; CORONA; METEOR) [18]. Both placebo subjects and statin users were included in this analysis. The interaction analysis followed the same statistical model as the primary GWAS, using the same phenotype and covariates, with the linear regression model this time also including extra terms encoding treatment allocation (statin vs placebo) main effect and the multiplicative interaction product term of treatment*SNP between treatment allocation. The interaction analysis was performed for the two lead SNPs reaching genome-wide significance (P < 5 ×10^−8^) from the primary GWAS meta-analysis. This interaction analysis was first conducted at cohort level and then results were meta-analyzed using METAL. For consistency with the primary GWAS meta-analysis, the interaction analysis only included individuals of European ancestry.

### 2.8. Reporting criteria for pharmacogenetic Loci

A locus is reported as a pharmacogenetic locus, only if the sentinel SNP meets both of the following criteria:

- A genome-wide significant (P < 5e-8) association from the GWAS for CRP statin response
- A significant (P-int<0.05) interaction result from the randomized statin v. placebo interaction analysis.

### 2.9. Secondary analysis in African ancestry participants

We assessed the two genome-wide significant lead SNPs identified in the European-ancestry GWAS within an African ancestry (AA) meta-analysis from the GIST consortium. This AA meta-analysis dataset included individuals who were verified to be of African ancestry according to both self-reporting and genetic verification. Three cohorts (PARC, JUPITER and MESA) contributed independent samples to the AA meta-analysis. The JUPITER participants were of South African ancestry, while MESA and CAP (part of PARC) participants were of African American ancestry. The GWAS statistical analysis model was exactly the same as for the European GWAS. The cohort-level GWAS results data from the three AA cohorts were combined into an AA meta-analysis using METAL (max N = 1461).

### 2.10. Lookups of other variants previously reported for statin response

We looked-up the results within this CRP statin response GWAS meta-analysis of five SNPs which have been reported in the literature from previously published GIST pharmacogenetics studies of statin response: four SNPs for LDL cholesterol response to statins (rs646776 at the *SORT1/CELSR2/PSRC1* locus; rs10455872 at the *LPA* locus; rs2900478 at the *SLCO1B1* locus; rs445925 at the *APOE* locus [6]); and one SNP for HDL cholesterol response to statins (rs247616 at the *CETP* locus [9]), in order to see if there is any support of these variants also influencing CRP response to statins.

## 3. Results

### 3.1. Cohort level GWAS

**Fig. 1.**
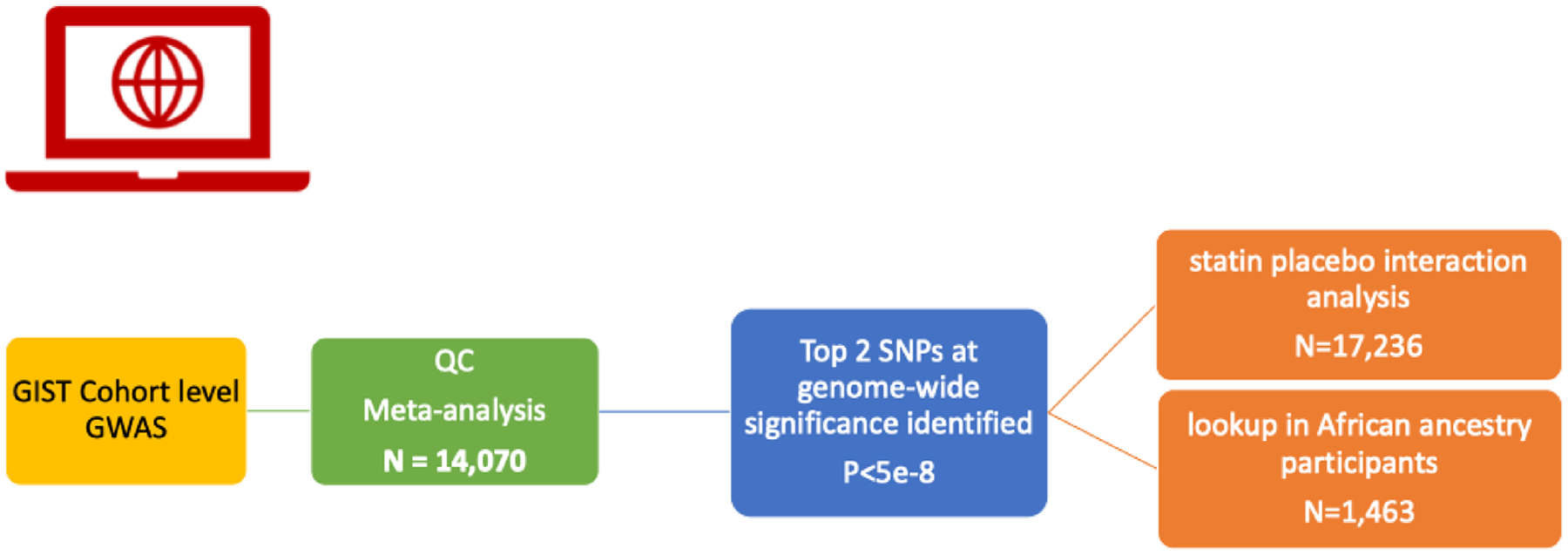
Study Design Flowchart.

The CARDS, FHS, JUPITER, MESA, PARC, PROSPER, and the MHI-AstraZeneca cohorts performed cohort level CRP response to statin therapy for a total sample size of 14,070 European ancestry participants treated with statins and with longitudinal CRP measurements before and after statin initiation. Sample sizes of each cohort and total N for the meta-analysis are shown in Table 1 and a study design flowchart is provided in Fig. 1. Cohort level information and demographics are shown in Supplementary Table 1.

### 3.2. GWAS meta-analysis and secondary analysis of results

**Fig. 2.**
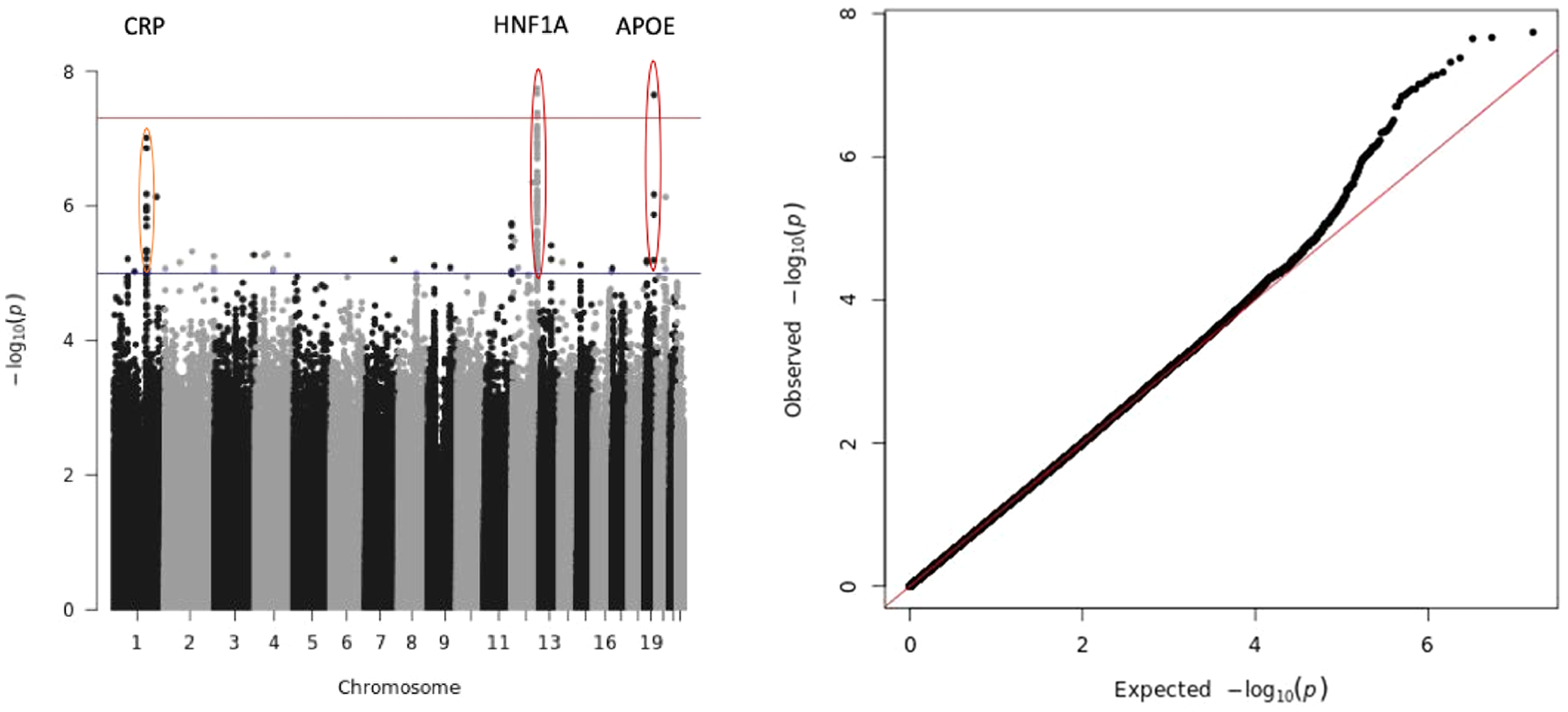
Manhattan plot and QQ plot of primary European GWAS meta-analysis. Filtered meta-analysis to N-studies ≥ 2. Lambda = 0.992.

Meta-analysis of all seven cohorts identified two loci achieving genome-wide significance (P < 5e-8): *APOE* (chromosome 19) and *HNF1A* (chromosome 12) (Fig. 2; Table 2). The next top hit was at the *CRP* gene locus (P = 9.7e-08), but this did not reach genome-wide significance. Detailed results are provided within Supplementary Table 2, showing association results for the sentinel centred SNP at all loci (1 Mb regions) reaching P < 1e-5 from the primary meta-analysis. Conditional analysis did not reveal any significant secondary signals, so we only report one signal from each of the two genome-wide significant loci.

**Fig. 3.**
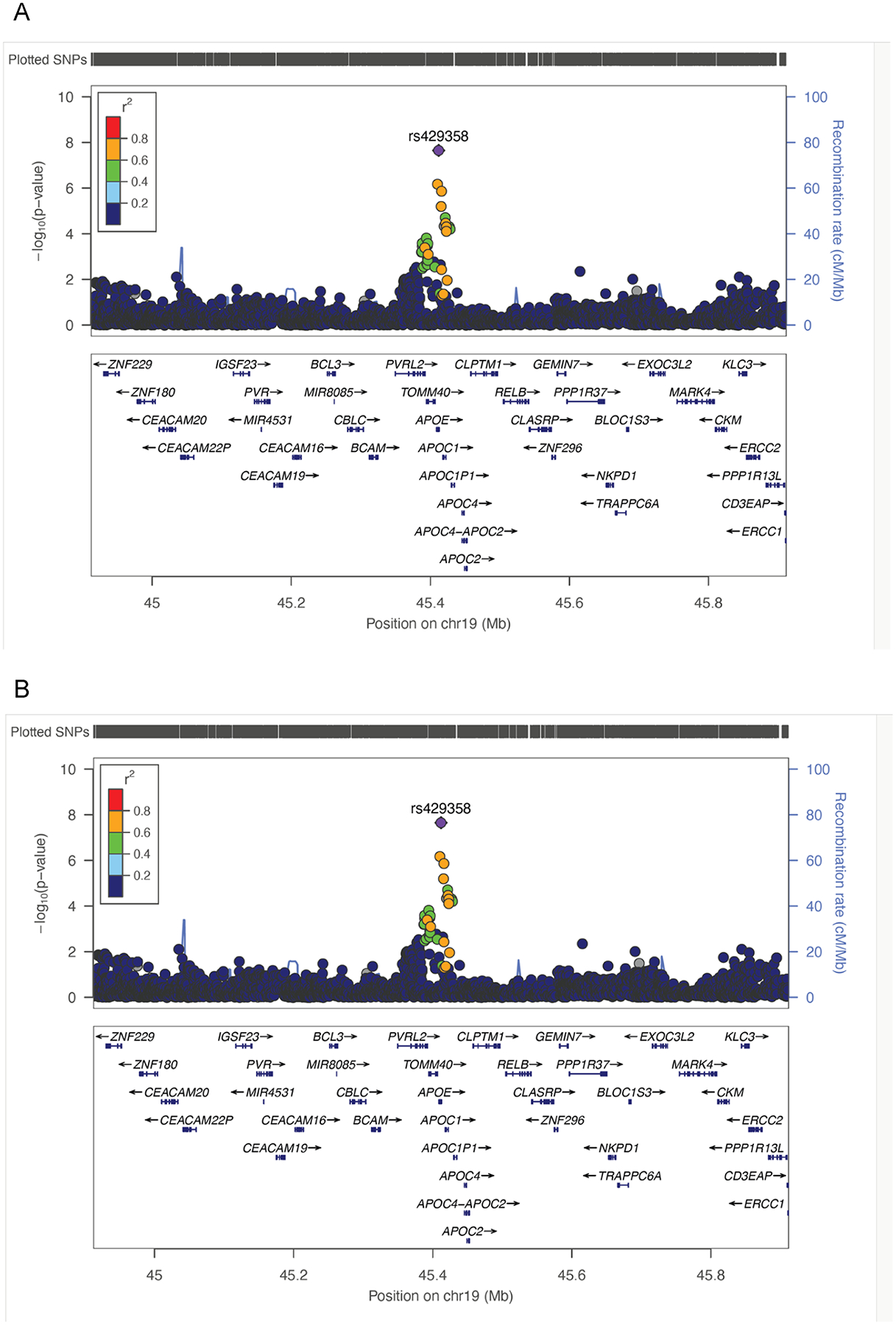
A, B. Locus Zoom plots for genome-wide significant loci from meta-analysis.

The most significantly associated variant at the *APOE* locus was rs429358 (Fig. 3A), which contributes to the *APOE* E4 haplotype and is a known risk loci for both dyslipidaemia and Alzheimer’s disease [19,20]. It is a c.388 T > C missense variant (MAF 0.12) leading to a n amino acid change p.Cys130Arg, known to be more common in African ancestry populations (MAF 0.27), and least common in Asian populations (MAF 0.09).

The most associated variant at the *HNF1A* locus was the intronic SNP rs11065384 (Fig. 3B) of MAF 0.31, which is in strong LD with the *HNF1A* missense SNP (rs1169288). The intronic T > C is known to be most common in African ancestry populations (EAF 0.90) and least common in Asian populations (EAF 0.59). The *HNF1A* locus is known to be associated with diabetes (both type 1 and type 2), cholesterol levels, and coronary artery disease [21–23]. In particular, the lead SNP rs11065384 here is in high LD (r^2^=0.88 from EUR ancestry 1000 G reference data) with the SNP rs1169288, which is a published variant for being associated with diabetes at genome-wide significance level [24].

**Fig. 4.**
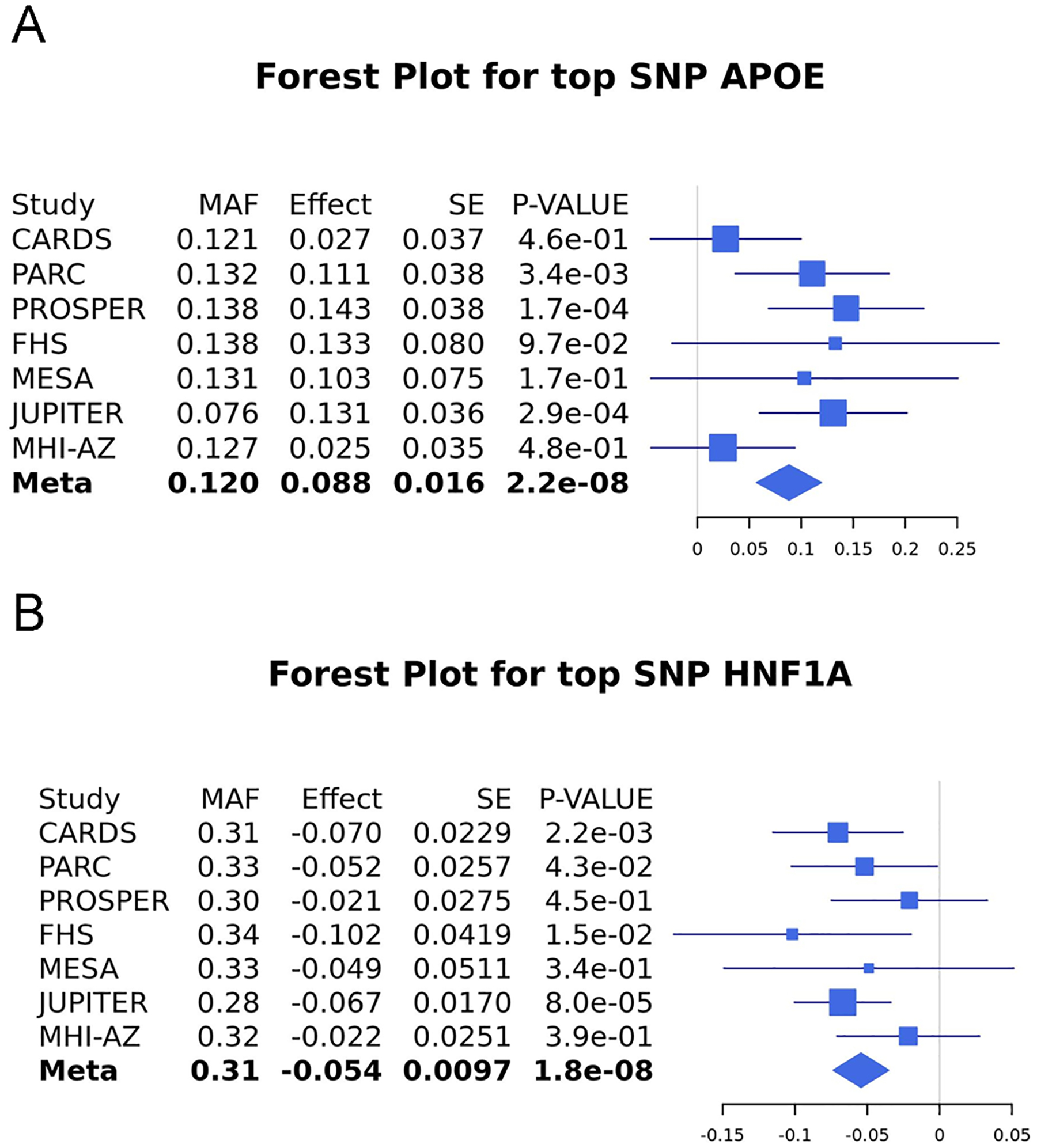
A,B – Forest plots genome-wide significant loci from the primary European GWAS meta-analysis. 4 A. Top SNP at *APOE*, rs429358. 4B. Top SNP at *HNF1A* rs11065384.

There was no between-study heterogeneity at the top three loci (P > 0.1) (Supplementary Figure 1), and forest plots for the lead SNPs at each of the two genome-wide significant loci show contribution to these signals from all seven studies within the meta-analysis (Fig. 4 A,B).

Both loci are also known to be associated with baseline CRP levels in the absence of statins [25–27].

PhenoScanner shows that the two significant loci from this CRP statin-response meta-analysis are also known to be significantly associated with many other traits of interest, as shown in Supplementary Table 3. The trait associations common to the two loci at *APOE* and *HNF1A* were CRP, lipids, haematological traits, and CVD. The *APOE* signal was also associated with dementia, Metabolic Syndrome, and other traits such as age-related macular degeneration and pulse rate. The *HNF1A* signal was also associated with hepatic traits.

The eQTL associations of particular interest include e.g. expression of *TOMM40* at the *APOE* locus in Lymphoblastoid cell lines tissue; and expression of *OASL* at the *HNF1A* locus in blood tissue. Both of these genes are known, from the literature and from GWAS Catalog, to affect LDL cholesterol and CRP [28](Supplementary Table 4).

### 3.3. Heritability analysis

We estimated the heritability (h^2^) using LDSC analysis to be h^2^= 1.77 % (SE = 0.0314), but this was a non-significant result with P = 0.286.

### 3.4. Statin x Placebo interaction analysis of two genome-wide significant lead SNPs

Neither of the two loci achieved a significant (P < 0.05) interaction analysis result. However, a suggestive association (P = 0.09) for interaction of the SNP rs429358 with randomized allocation to statin or placebo within samples derived from clinical trials provides promising evidence to support our hypothesis for there being a pharmacogenetic effect at *APOE* beyond genetic effects on baseline CRP levels (which were adjusted for by inclusion of baseline CRP as a covariate in the GWAS analysis). Results for the interaction meta-analysis are shown in Table 3. There was no support however for the lead *HNF1A* SNP from the interaction analysis (P = 0.81).

### 3.5. Association of two genome-wide significant lead SNPs within an African ancestry meta-analysis

The meta-analysis of the sentinel SNPs at the two genome-wide significant loci from the primary European GWAS meta-analysis was performed on 1463 participants of African ancestry (from CAP (a sub-study within PARC), JUPITER and MESA). Neither of the SNPs showed significant evidence (P > 0.05) of support within African ancestry, although the effect estimate of the *APOE* variant was in a concordant direction in European vs African ancestry analyses, with similar effect size (0.058 in African ancestry vs 0.088 in European ancestry). Results are shown in Supplementary Table 5.

### 3.6. Lookups of other variants previously reported for statin response

None of the five SNPs reported from previously published GIST pharmacogenetics studies of statin response to LDL or HDL cholesterol show any evidence (all have P > 0.1) from this CRP GWAS meta-analysis of also being associated with CRP response to statins (Supplementary Table 6).

## 4. Discussion

We report here that common variants at *APOE* and *HNF1A* are found to be associated with CRP response to statins at genome-wide significance level from a large GWAS meta-analysis. However, we cannot confirm pharmacogenetic effects at either of these loci, as neither of these loci achieved significant interaction results (P < 0.05) in the placebo vs treatment interaction analysis, although this secondary analysis may have been underpowered. Nevertheless, the suggestive interaction result (P = 0.09) for APOE E4 still provides promising support for a potential pharmacogenetic effect, which warrants further follow-up.

The *APOE* gene is also known to be associated with LDL cholesterol response to statins [6]. The SNP rs445925 at the *APOE* locus, which is in LD (r^2^=0.66 from EUR-1000G data) with the SNP rs7412, contributing to the *APOE* E2 haplotype, has been previously reported for association with LDL cholesterol response to statins [6]. Furthermore, in unpublished recent work from the GIST consortium, the lead SNP rs429358 from this CRP statin response GWAS meta-analysis here, contributing to the *APOE* E4 haplotype, has also been identified to be associated with LDL cholesterol response to statins [29]. Hence this latest GIST GWAS meta-analysis of LDL statin response using 1000 G imputed data shows that both the *APOE* E2 haplotype and *APOE* E4 haplotype signals are associated with LDL cholesterol response to statins. We note that these are independent signals of association, with the rs445925 and rs7412 SNPs not being in LD with the lead SNP rs429358 here (r^2^<0.02 from EUR-1000G data). Instead, this CRP statin response GWAS shows that CRP response to statins is only influenced by rs429358 (at the *APOE* E4 haplotype), with no effects at rs7412 (at the *APOE* E2 haplotype). This firstly shows interesting specificity of pharmacogenomic mechanisms at the *APOE* locus. Secondly, this demonstrates evidence of distinct genetic architecture between LDL cholesterol response to statins vs CRP response to statins, as also illustrated by the lack of significance from this CRP statin response GWAS for any of the other previously reported pharmacogenetic statin response variants (Supplementary Table 6). This is consistent with the JUPITER CRP statin response study, which also found no effect for CRP-change for the rs7412 SNP at the *APOE* E2-haplotype within their LDL cholesterol response candidate gene analysis, though the rs429358 SNP at the *APOE* E4-haplotype identified in the CRP statin response GWAS meta-analysis was not tested within their candidate SNP analyses ^10^. The minor allele C of the rs429358 SNP at *APOE*, which is known to be associated with a decrease in baseline CRP levels, is shown in this CRP statin response GWAS to be associated with an improved reduction in CRP from statin therapy. Therefore, the minor allele C of the rs429358 SNP shows concordance in direction between effect on baseline CRP and effect from this study on CRP response to statins. Similarly, knowing that this SNP is associated with both CRP and LDL, there is also concordance in direction between effect on baseline LDL and LDL statin response for the rs429358 SNP at the *APOE* E4 haplotype, with the minor allele C known to be associated with an increase in baseline LDL levels, and shown in our latest unpublished LDL statin response GWAS to be associated with a poorer LDL statin response [29]. However, it is interesting to note that the minor allele C of this rs492358 SNP is therefore working in opposite directions on CRP vs LDL, decreasing CRP levels in response to statins, but associated with a poorer LDL-cholesterol response to statins. For comparison, for LDL association only, the minor allele T of the rs7412 SNP at the *APOE* E2 haplotype concordantly improves both baseline LDL levels and LDL statin response.

In contrast, the *HNF1A* locus, which reached genome-wide significance in the GWAS analysis, has never been reported for any other statin response associations. Variation in the *HNF1A* gene was tested as a CRP-associated candidate gene in the JUPITER study, where it reached nominal significance but was not considered significant after accounting for multiple testing of other candidate variants using Bonferroni correction.

The two signals that achieved genome-wide significance from the GWAS meta-analysis (*APOE* and *HNF1A*) are of substantial mechanistic and clinical interest. The association between *APOE* and Alzheimer’s disease risk is well-known [30]. There has also been clinical interest in the effects of statin treatment on dementia. Linking this together, there have also been extensive efforts to characterize the relationship between statin use and dementia outcomes, with re-analysis of some clinical trials along with observational data suggesting a benefit within statin-users in Alzheimer’s disease onset and progression specific to *APOE* E4 homo-zygotes only, who are known to have a heightened risk of Alzheimer’s disease [31]. A more recent study of 1725 participants from the Alzheimer’s Disease Neuroimaging Initiative used a mediation model stratified by statin use and *APOE* E4 alleles to conclude that *APOE* E4 carriers had better cognitive outcomes if treated with a statin, and that indeed statin use compensated for the increased risk to E4 carriers as compared with non-carriers [32]. Such examples in the literature demonstrate a link between the *APOE* E4 endotype of Alzheimer’s and a benefit of statins treatment on dementia outcomes. However, it has always been unclear what is driving this relationship, and the underlying mechanisms remain unknown. For the first time, within a hypothesis-free GWAS approach analysis, we suggest a potential pharmacogenetic association between *APOE* and CRP response to statins, suggesting a link between *APOE* carrier status, statin treatment and the influence on inflammation, such as the CRP biomarker. By identifying an effect of *APOE* genotype on CRP change with statin treatment, the results from this CRP statin response GWAS are therefore hypothesis-generating and motivate the consideration of whether the benefit of statins on dementia outcomes may be mediated perhaps via a CRP pathway and due to an inflammation mechanism. It is particularly exciting that this hypothesis-free GWAS has identified *APOE* as a locus with a genome-wide significant association for CRP statin response, whereas, in contrast, all previously discussed examples from the literature investigating a link between *APOE* E4 endotype Alzheimer’s and a benefit of statins treatment on dementia outcomes originate from candidate-gene based analyses, stemming from the a-priori interest from the known association between *APOE* E4 and Alzheimer’s. Currently, the attempts in the literature to draw links between CRP, statins and *APOE*, have led to inconsistent findings. As noted above, the Alzheimer’s Disease Neuroimaging Initiative study deduced that “the effects of statins on CRP are moderated by *APOE* E4 alleles” [32]. These conclusions contrast with an earlier study of CRP in atherosclerotic patients from the Age Gene/Environment Susceptibility (AGES)-Reykjavik Study, which found that patients with *APOE* E4 had lower CRPs then counterparts on and off statin and that this effect was independent of statin use [33]. The results from this CRP statin response GWAS here provide a reason to re-consider this hypothesis. Indeed, the agnostic approach here to genomics of CRP in response to statin provides orthogonal evidence for an inflammatory mechanism as a potential mediator of the observed *APOE* E4 specific statin benefit in dementia outcomes and may answer some of these remaining questions, provide further support for the studies reported above and strengthen motivation to investigate the benefit of statins for dementia in known *APOE* E4 carriers. However, it is important that this be treated as a lead for further investigation rather than a definitive pharmacogenomic response, given that the interaction analysis was only suggestive but did not meet standard significance thresholds at this locus.

This is a timely topic as regulators are currently examining multiple new monoclonal antibodies that target beta-amyloid. The risk of an adverse drug reaction, amyloid-related imaging abnormalities (ARIA), is increased and potential benefit decreased in *APOE* E4 homozygote [34]. Therefore, there is consideration currently being given to test for *APOE* E4 if there is an indication for one of these medications [35]. As this is not merely a biomarker for adverse drug reaction risk but also an independent risk prediction locus for both Alzheimer’s and dyslipidemia, this may mean cascade testing and identification of increasing numbers of patients with *APOE* E4 pre-symptomatically [19,36]. Thus, it is important to revisit discussion of therapeutic potential of statins to decrease dementia risk in *APOE* carriers. If testing for *APOE* status in the context of these monoclonal antibodies proceeds, we would learn the *APOE* E4 status of many more individuals than we do currently in routine clinical practice and therefore have a timely opportunity to investigate a pharmacologic approach to optimize therapy for this *APOE* E4 endotype of Alzheimer’s Disease.

Furthermore, it is important to consider the hypothesis that the *APOE*-E4-related statin effect on CRP could also be relevant to cardiovascular disease outcomes, as well as to dementia, as suggested by results from the Scandinavian Simvastatin Survival Study, where a differential effect of statin on CVD mortality was identified in *APOE*-E4 carriers independent of lipids [37].

The *HNF1A* locus is also of obvious interest given the known association of statins with increased diabetes incidence [38]. *HNF1A* mutations, and in particular the SNP *rs1169288 (in LD with the lead SNP rs11065384 from this CRP statin response GWAS)*, are known to be associated with monogenic diabetes, formerly known as maturity-onset diabetes of the young 3, which is a condition particularly responsive to sulphonylureas [39]. *HNF1A* associated monogenic diabetes is known to be associated with lower CRP that can be used diagnostically to help differentiate type 1 and 2 diabetes [40]. Furthermore, *HNF1A* variants, also contribute to the susceptibility to type 2 diabetes [41]. However, the interaction analysis did not support a placebo vs drug effect for this *HNF1A* variant, therefore it is likely that this hit is not indicative of a true pharmacogenomic effect.

The notable lack of well-known pharmacokinetic genes implicated in the metabolism of statins among the results of this study point to a pharmacodynamic (how the drug affects the body) rather than pharmacokinetic effect (how the body affects the drug) of statins in reducing inflammation. The way the body metabolises and excretes statins would be expected to lead to well understood pharmacokinetic signals if the level of exposure of active metabolites were mediating CRP response. It also suggests this is not likely to be a dose-dependent phenomena, at least within the range of doses examined by studies contributing to the current analysis.

The secondary analysis in individuals of African ancestry was likely underpowered given the dynamic nature of the CRP biomarker involved, the ancestral heterogeneity of the African participants, and the small number (n = 1463) of participants in the AA meta-analysis. The LD structure may also be different in African individuals, leading to a similar signal from a different SNP in the region, which we did not look for due to the low number of African participants. However, the varying allelic frequency of the lead SNPs across diverse ancestry groups suggest that a pharmacogenomics effect of statins on CRP, if, validated, may be of a different population prevalence in African or Asian ancestry populations, both of which are under-represented in therapeutic trial cohorts.

### 4.1. Strengths and limitations

CRP is a dynamic biomarker which leads to high noise-to-signal ratio in assessments. A similar approach to this study with a larger number of participants included in the future could have more potential to detect more significant signals. A major strength of this study is its access to non-opportunistic longitudinal CRP data in individuals before and after statin exposure, which is a rare and valuable resource. However, because the physiological CRP value is near zero, representing a finite lower limit, it restricts our ability to demonstrate a significant decrease in CRP levels for individuals starting at a low baseline. This limitation introduces a potential bias which is unavoidable in this study. While JUPITER inclusion criteria included CRP baseline criteria (CRP of at least 2.0 mg per litre), the other studies in this GIST meta-analysis did not [2].

This GWAS analysis of CRP change was adjusted for the baseline CRP value to better account for bias due to baseline levels and to regress out any residual signals from genetic variants associated only with baseline CRP levels. However, we also acknowledge that potential biases can occur from baseline-adjusted models [42]. The second requirement of a significant statin v. placebo interaction analysis result is therefore important, in addition to the significant GWAS association. We also considered the statin v. placebo interaction analysis result for the peri-significant *CRP* locus from this GWAS meta-analysis. Despite this *CRP* locus being highly significant (but not reaching genome-wide significance) in this CRP statin response GWAS, and of course the *CRP* locus being associated with baseline CRP levels, the interaction analysis result for the *CRP* locus was non-significant (P > 0.1). This provides a good negative control example, that non-significant results from the statin v. placebo interaction analysis can help to eliminate any loci from the CRP statin response GWAS, which do not represent pure pharmacogenetic effects, in addition to genetic effects on baseline CRP levels. Similarly, it is reassuring indeed in the first place that this *CRP* locus did not achieve the genome-wide significance threshold in this CRP statin response GWAS, indicating some success in the baseline-CRP covariate adjustment in this pharmacogenetic GWAS model too. In contrast, the genome-wide significant result for the *APOE* locus from this CRP statin response GWAS, together with the suggestive interaction result for the *APOE* locus provides promising support for the hypothesis of a pharmacogenetic effect at this locus.

Overall, the genome-wide significant results for both the *APOE* and *HNF1A* loci from this GWAS of CRP statin response warrant further investigation. To ensure this GWAS was sufficiently powered, all datasets from the GIST consortium with available CRP measurements were meta-analysed together into a single-stage GWAS analysis, leaving no further studies available to use for a sufficiently powered replication stage dataset. It will be important to validate these pharmacogenetic associations within independent datasets in the future.

It will also be important, in future studies, to confirm whether there is indeed a true pharmacogenetic effect at the *APOE* locus, following up upon the suggestive interaction result here. If a pharmacogenetic effect for CRP statin response can be confirmed for *APOE*, this knowledge will be very timely, considering other ongoing investigations.

## Supplementary Material

SupplementaryDOC

## Figures and Tables

**Table 1:** Sample sizes of each cohort and total N for the meta-analysis.

Study Cohort	N participants
CARDS	1005
FHS	1114
JUPITER	4167
MESA	548
PARC	1858
PROSPER	2383
MHI-AstraZeneca cohort	2995
**TOTAL**	**N = 14,070**

**Table 2:** Primary European GWAS meta-analysis genome top loci.

MarkerName	rsID	Gene	A1	A2	Freq1	Effect	StdErr	P value	N	MAF	N-Studies
19:45411941	rs429358	*APOE*	T	C	0.880	0.088	0.015	2.2e-8	14,070	0.120	7
12:121423285	rs11065384	*HNF1A*	T	C	0.307	− 0.054	0.010	1.8e-8	14,070	0.307	7

**Table 3:** Meta-analysis of SNP*treatment interaction effects using placebo-controlled trial data at the genome-wide significant SNPs.

MarkerName	A1	A2	Freq1	Effect	StdErr	P value	N
19:45411941	T	C	0.902	−0.0666	0.0388	0.086	17,236
12:121423285	T	C	0.289	−0.0061	0.0250	0.806	17,236

## Data Availability

At the time of publication, the full GWAS summary statistics from this primary CRP statin response GWAS meta-analysis data in EUR ancestry, will be made publicly available via GWAS catalogue

## Appendix A. Supporting information

Supplementary data associated with this article can be found in the online version at doi:10.1016/j.phrs.2024.107575.
