# Supplementary material for: GWAS of CRP response to statins further supports the role of APOE in statin response: A GIST consortium study
Source: Pharmacol Res. Author manuscript; Available in PMC 2026 May 31. (PMC13222656; doi:10.1016/j.phrs.2024.107575)

**Supplementary Table 1A: Supplementary Data from each Participating Cohort: General Study Information**

| **Study** | **N** | **Type** | **Cohort Full name** | **Website** | **Description** | **Ethnicity & Origin** | **Study Design** | **Refs [PMID]** |
| --- | --- | --- | --- | --- | --- | --- | --- | --- |
| MHI-AZ | 2,995 | trials | Montreal Heart Institute - AstraZeneca cardiometabolic cohort | N/A | Collection of randomized clinical trials for cardiometabolic indication | Similarity to CEU cluster by PCA | RCT | 17984166, 19332456, 21446892, 24401221, 17384434, 18402897, 25660356 |
| CARDS | 1,174 | trial | Collaborative Atorvastatin Diabetes Study | N/A | All T2D cases | European from UK | RCT | 18777490; 25899452 |
| FHS | 1,353 | cohort | Framingham Heart Study | <https://www.framinghamheartstudy.org/> | Population-based Sample in Framingham, MA | EUR Ancestry living in Framingham, MA | Population-based sample from Framingham in 1948 | 14025561; 14819398; 1208363; 474565; 17372189 |
| JUPITER | 7,763 (EUR); 1,520 (AA) | trial | Justification for the Use of Statins in Prevention: an Intervention Trial Evaluating Rosuvastatin | <https://clinicaltrials.gov/study/NCT00239681> | A trial of rosuvastatin (20mg/day) in primary prevention of cardiovascular events among individuals with LDL-C < 130mg/dl and hsCRP >= 2.0 mg/l. | Genetic analysis verified EUR ancestry (Largely from Americas or Europe) & South African Black ancestry (S.Africa) | International, multi-center RCT | 18997196, 22331829, 22230323 |
| MESA | 548 | cohort | Multi-Ethnic Study of Atherosclerosis | <https://www.mesa-nhlbi.org/> | population-based samples | European from US | longitudinal study; population-based samples | 12397006 |
| PROSPER | 2,550 | trial | PROspective Study of Pravastatin in the Elderly at Risk | N/A | Subjects with a history of Cardiovascular disease or a high risk for CVD | European | Randomised control clinical trial | 12457784 |
| CAP | 562 | trial | Cholesterol and Pharmacogenetic Study (part of PARC) | <https://clinicaltrials.gov/ct2/show/NCT00451828> | 6-wk non-randomized, open label study of simvastatin 40 mg/day in 335 AA and 609 EUR volunteers; At screening visit: age 30+, TC 160-400 mg/dl, TG <400 mg/dl, FG <126 mg/dl | European and some Middle Eastern (including Ashkenazi Jews) Americans | 40 mg/day 6 week simvastatin trial | 16516587 |
| PRINCE | 1,242 | trial | PRavastatin Inflammation CRP Evaluation (part of PARC) | N/A | 40mg/day pravastatin for 24 weeks; all got drug in secondary prevention arm; randomized to drug/placebo in primary prevention arm | Self-identified white | randomized control primary prevention; open label secondary prevention | 11376301 |

**Supplementary Table 1B: Supplementary Data from each Participating Cohort: Genotyping & Quality Control (QC)**

| **Cohort** | **Ancestry** | **GWAS Genotyping Chip Array** | **Calling Algorithm** | **SNP MAF** | **SNP Call Rate** | **SNP QC HWE_p** | **Sample Call Rate** | **Sample QC Other (IBS clustering, heterozygosity)** | **1000G Panel** | **Impute Software** | **Relateds (yes/no)** | **Related adj method** | **Population stratification assessment & adjustment** | **Analysis software** |
| --- | --- | --- | --- | --- | --- | --- | --- | --- | --- | --- | --- | --- | --- | --- |
| CARDS | EUR | Perlegen | Perlegen 6 | >0.1% | 98% | 1e-6 | 99% | excl duplicates; gender mismatch, population outliers | 1000G  Phase 1v3 March 2012 | IMPUTE | no | N/A | PCs calculated by Eigenstrat | SNPTEST |
| FHS | EUR | Affymetrix 250K Sty, 250K Nsp & MIPS 50K, Gene Centric | BRLMM | > 0% | 97% | 1e-6 | 97% | heterozygosity (mean+5 SD); >1000 Mendelian errors | 1000G Phase 1v3 Nov 2010 | Minimac | yes | Linear Mixed Effects Model with Kinship | PCs calculated by Eigenstrat | R program lmekin |
| MESA | EUR | Affymetrix Genome-Wide Human SNP Array 6.0 | Birdseed | >5% | 95% | 1e-6 | 95% | excl duplicates and unresolved gender mismatches; SNPs with het > 53% | 1000G Phase 1v3 March 2012 | IMPUTE | no | NA | Ancestry outliers by PCA | RVTEST |
| PARC | EUR | Illumina Human 317K and 610_Quad | Beadstudio | >0% | 99% (317K); 95% (610K) | 1e-4 | 99% | 6 gender mismatches present in data | 1000G Phase 1v3 March 2012 | IMPUTE | no | NA | adj for 5 PCs, excl some probable AA & Hispanics | SNPTEST V2 |
| PROSPER | EUR | Illumina Human 660_Quadv1 | Beadstudio | >0.01% | >=98% | N/A | >97.5% | excluded duplicates; gender mismatch | 1000G Phase 1v3 March 2012 | IMPUTE | no | 3SD | Ancestry outliers by PCA | SNPTEST |
| MHI-AZ | EUR | Illumina Infinium Multi-Ethnic Global Array (MEGA) Consortium v2 BeadChip | Beeline v2.0 | 0% | 98% | 3.3e-8 | 98% | excl duplicates, possible contaminated samples, gender mismatch, population outliers | TOPMed r2 | Minimac4 v1.7.3 | no | NA | Ancestry outliers by PCA, adjusted for 10 PCs | genetest v0.6.0 |
| JUPITER | EUR; AA | Illumina Omni 1M | Genome Studio (v. 1.6.2) |  | >99% | 1e-6 | >95% | sex check, unrelated only, ancestry strata verified by MDS clustering | 1000G Phase 1v3 March 2012 | Minimac/University Michigan server | no | NA | adjusted for top 10 PCs | ProbABEL |
| MESA | AA | Affymetrix Genome-Wide Human SNP Array 6.0 | Birdseed | >5% | 95% | 1e-6 | 95% | excl duplicates and unresolved gender mismatches; SNPs with het >53% | 1000G Phase 1v3 March 2012 | IMPUTE | no | NA | Ancestry outliers by PCA | RVTEST |
| CAP | AA | Illumina Omni2.5 +exome, Illumina Cardio-metabochip, Illumina Immunochip | Beadstudio | >0% (per array) | 98% (per array) | 1e-5 (per array & overall) | 98% (per array) | excluded gender mismatches | 1000G phase 3v5 | MaCH-Admix | no | excluded if pi_hat >.125 | excluded ancestry outliers; adjusted for 4 ancestry PCs | mach2qtl |

**Supplementary Table 1C: Supplementary Data from each Participating Cohort: Summary Descriptive Statistics**

| **Cohort** | **Ancestry** | **N analysed** | **Male** | **Age (at baseline or DNA)** | **Age range** | **BMI, kg/m2** | **Diabetes** | **Hypertension** | **history of MI at baseline** | **history of CHD at baseline** |
| --- | --- | --- | --- | --- | --- | --- | --- | --- | --- | --- |
|  |  |  | **N (%)** | **mean (SD)** |  | **mean (SD)** | **N (%)** | **N (%)** | **N (%)** | **N (%)** |
| CARDS | EUR | 1194 | 632(53) | 61.6(8.2) | 40-76 | 28.7(3.6) | 1194(100%) | 1038 | 0% | 0% |
| FHS | EUR | 1484 | 819 (55.2) | 62.4 (9.7) | 28-92 | 29.3 (5.3) | 187 (12.6) | 871 (58.7) | 136 (9.2) | 136 (9.16) |
| MESA | EUR | 548 | 264 (48) | 64.6 (9.1) | 45-88 | 28.7 (5.5) | 69 (12.6) | 310 (56.6) | NA | NA |
| **PARC (CAP+PRINCE)** | EUR | **1865** | **1306(70)** | **61.7(13.7)** | **26-100** | **28.6(5.4)** | **276(15)** | **933(50)** | **425(23)** | **N/A** |
| *CAP* | EUR | *565* | *301(53)* | *54.5(12.6)* | *30-88* | *27.7(5.4)* | *11(2)* | *405(70)* | *3(0.5)* | *N/A* |
| *PRINCE* | EUR | *1300* | *1005(77)* | *64.8(13.0)* | *26-100* | *29.0(5.3)* | *265(20)* | *528(41)* | *422(32)* | *N/A* |
| PROSPER | EUR | 2550 | 1228 (48) | 75.4 (3.4) | 70-83 | 26.8 (4.1) | 256 (10) | 1592 (62) | 338 (13) | N/A |
| MHI-AZ | EUR | 2995 | 2088 (69.7) | 66.1 (10.5) | 22-90 | 28.06 (5.29) | 786 (26.24%) | 1976 (65.98%) | 996 (33.26%) | 1519 (50.72%) |
| JUPITER-EA | EUR | 7763 | 5268 (68) | 66.0 (7.7) | 50-93 | 29.4 (7.7) | 30 (0.4) | 4353 (56) | 0 | 0 |
| JUPITER-AA | AA | 1520 | 760 (50) | 66.2 (7.9) | 50-94 | 28.4 (7.9) | 3 (0.2) | 1032 (0.68) | 0 | 0 |
|  |  |  |  |  |  |  |  |  |  |  |
| CAP | AA | 314 | 153(49) | 54.2(12.0) | 31-88 | 30.2(6.6) | 25(8) | 252(80) | 8(2.5) | N/A |
| MESA | AA | 283 | 140 (49) | 63.1 (8.7) | 45-83 | 30.2 (5.5) | 95 (33.6) | 222 (78.5) | NA | NA |

**Supplementary Table 1D: Supplementary Data from each Participating Cohort: Statin Information**

| **Cohort** | **Ancestry** | **N (analysed)** | **Type of statin** | **%Atorva** | **%Simva** | **%Prava** | **%Lova** | **%Rosuva** | **%Fluva** | **Statin dose (mg/day)** | **CRP off-treatment (mg/L); Mean (SD)** | **CRP on-treatment (mg/L) (mean of multiple measures); Mean (SD)** | **Follow-up time between off-tmt & on-tmt measures (months); Mean (SD)** |
| --- | --- | --- | --- | --- | --- | --- | --- | --- | --- | --- | --- | --- | --- |
| CARDS | EUR | 1194 | Atorvastatin | 100% | 0% | 0% | 0% | 0% | 0% | 20mg | 1.3 (IQR 0.6, 3.1) | 1.2 (IQR 0.5, 2.9 | 3·9 years (IQR 3·0–4·7 |
| FHS | EUR | 1355 | Any lipid lowering drug | N/A | N/A | N/A | N/A | N/A | N/A | Any lipid lowering drug | N/A | N/A | 63.5 (21.9) |
| MESA | EUR | 548 | A (N=226), F (N=6), L (N=32), P (N=65), R (N=33), S (N=185) | 41% | 33% | 12% | 6% | 6% | 1% | A (16.2), F (63.3), L (24.2), P (31.7), R (11.1), S (28) | 3.35 (4.55) | 2.67 (4.62) | 33.7 (18.0) |
| **PARC (CAP+PRINCE)** | EUR | **1865** | **P (N=1300), S (N=565)** |  |  |  |  |  |  | **40** |  |  | **12 wks (P), 6 wks (S)** |
| *CAP (part of PARC)* | EUR | *565* | *simvastatin* | 0% | 100% | 0% | 0% | 0% | 0% | *40* | 2.12(3.08) | 2.11(3.50) | *6 weeks* |
| *PRINCE (part of PARC)* | EUR | *1300* | *pravastatin* | 0% | 0% | 100% | 0% | 0% | 0% | *40* | 0.41(0.67) | 0.38(0.84) | *12 weeks* |
| PROSPER | EUR | 2550 | Pravastatin | 0% | 0% | 100% | 0% | 0% | 0% | 40 | 5.99 (11.46) | 3.82 (6.00) | 29.5 (9.2) |
| MHI-AZ | EUR | 2995 | Atorva (N=202)  Rosuva (N=2634)  Simva (N=159) | 6.74% | 5.31% | 0% | 0% | 87.95% | 0% | R (16.02)  A (80)  S (60.62) | 4.27 (10.24) | 3.38 (9.18) | 5.9 (6.98) |
| JUPITER | EUR | 4,167 | rosuva | 0% | 0% | 0% | 0% | 100% | 0% | 20 | 5.4 (7.7) | 3.9 (7.7) | 12 months |
| JUPITER | AA | 858 | rosuva | 0% | 0% | 0% | 0% | 100% | 0% | 20 | 9.7 (7.9) | 7.9 (7.9) | 12 months |
| MESA | AA | 283 | A (N=95), F (N=7), L (N=32), P (N=25), R (N=13), S (N=111) | 34% | 39% | 9% | 11% | 5% | 2% | A (19.1), F (62.9), L (25.2), P (32.3), R (9.0), S (28.4) | 4.38 (6.39) | 3.96 (6.97) | 33.9 (18.8) |
| CAP | AA | 314 | simvastatin | 0% | 100% | 0% | 0% | 0% | 0% | 40 | 3.30(7.01) | 2.95(7.33) | 4&6 weeks |

**Supplementary Table 2.** **Further results of primary European GWAS meta-analysis showing the sentinel SNP at all loci (1Mb) reaching P<1e-5**

| **MarkerName** | **rsID** | **A1** | **A2** | **Freq1** | **Effect** | **StdErr** | **P value** | **HetP** | **N** | **NStudies** |
| --- | --- | --- | --- | --- | --- | --- | --- | --- | --- | --- |
| 12:121423285 | rs11065384 | T | C | 0.31 | -0.054 | 0.010 | 1.82E-08 | 0.48 | 14,070 | 7 |
| 19:45411941 | rs429358 | T | C | 0.88 | 0.088 | 0.016 | 2.25E-08 | 0.12 | 14,070 | 7 |
| 1:159676011 | rs2808628 | A | G | 0.32 | -0.051 | 0.010 | 9.71E-08 | 0.68 | 14,070 | 7 |
| 12:97709871 | rs184343086 | A | G | 0.98 | 0.271 | 0.054 | 4.53E-07 | 0.95 | 4,548 | 3 |
| 1:207323414 | rs4339867 | T | C | 0.03 | 0.192 | 0.039 | 7.34E-07 | 0.24 | 9,355 | 5 |
| 20:42413027 | 20:42413027 | D | I | 0.88 | 0.104 | 0.021 | 7.40E-07 | 0.93 | 6,360 | 4 |
| 11:131914806 | rs533991 | T | C | 0.61 | -0.044 | 0.009 | 1.84E-06 | 0.78 | 14,070 | 7 |
| 12:12668962 | rs150860045 | A | G | 0.38 | -0.054 | 0.012 | 3.30E-06 | 0.67 | 9,903 | 6 |
| 13:77067297 | rs139706061 | A | T | 0.11 | 0.085 | 0.019 | 3.89E-06 | 0.74 | 9,355 | 5 |
| 2:131762905 | rs7579742 | A | G | 0.90 | -0.090 | 0.020 | 4.81E-06 | 0.40 | 8,789 | 5 |
| 4:59628770 | rs12716066 | T | C | 0.25 | -0.097 | 0.021 | 5.18E-06 | 0.06 | 6,360 | 4 |
| 3:191844140 | rs870808 | T | C | 0.69 | 0.044 | 0.010 | 5.40E-06 | 0.58 | 14,070 | 7 |
| 4:158444726 | rs147750317 | T | C | 0.97 | -0.176 | 0.039 | 5.43E-06 | 0.91 | 9,903 | 6 |
| 4:44662095 | rs6842865 | T | C | 0.06 | 0.126 | 0.028 | 5.57E-06 | 0.93 | 6,908 | 5 |
| 2:240101713 | rs3791536 | A | G | 0.19 | 0.056 | 0.012 | 5.66E-06 | 0.45 | 13,065 | 6 |
| 1:66231771 | rs12131840 | T | C | 0.13 | -0.062 | 0.014 | 6.20E-06 | 0.91 | 14,070 | 7 |
| 7:136857683 | rs833006 | T | C | 0.04 | -0.145 | 0.032 | 6.33E-06 | 0.94 | 9,355 | 5 |
| 19:10465832 | rs8108236 | A | G | 0.09 | -0.094 | 0.021 | 6.51E-06 | 1.00 | 8,789 | 5 |
| 20:32721299 | rs34618271 | C | G | 0.65 | 0.055 | 0.012 | 6.55E-06 | 0.50 | 9,355 | 5 |
| 14:35477013 | 14:35477013 | D | I | 0.52 | -0.072 | 0.016 | 6.93E-06 | 0.07 | 4,502 | 3 |
| 2:73388458 | rs114596492 | T | C | 0.97 | 0.205 | 0.046 | 6.96E-06 | 0.91 | 6,406 | 4 |
| 15:37664874 | rs36035696 | D | I | 0.37 | 0.053 | 0.012 | 7.59E-06 | 0.78 | 9,355 | 5 |
| 9:29020053 | rs10968884 | A | C | 0.86 | 0.059 | 0.013 | 7.79E-06 | 0.62 | 13,522 | 6 |
| 9:108380004 | rs10816284 | A | G | 0.42 | -0.040 | 0.009 | 8.26E-06 | 0.15 | 14,070 | 7 |
| 12:29200329 | rs117706837 | A | G | 0.02 | 0.336 | 0.075 | 8.30E-06 | 0.81 | 3,497 | 2 |
| 17:248275 | rs113498980 | D | I | 0.03 | 0.204 | 0.046 | 8.45E-06 | 0.70 | 5,246 | 3 |
| 2:1788623 | rs6748017 | T | G | 0.77 | 0.051 | 0.011 | 8.49E-06 | 0.53 | 13,522 | 6 |
| 4:89335667 | rs6850385 | T | C | 0.27 | -0.045 | 0.010 | 8.66E-06 | 0.13 | 14,070 | 7 |
| 1:100753180 | rs72730117 | A | T | 0.04 | 0.133 | 0.030 | 9.46E-06 | 0.12 | 9,903 | 6 |
| 16:85437603 | rs117017591 | A | G | 0.94 | -0.126 | 0.029 | 9.64E-06 | 0.37 | 8,789 | 5 |

**Supplementary Table 3. PhenoScanner-derived GWAS trait associations for genome wide significant SNPs from the GWAS meta-analysis.** (Signals in common from the two SNPs are noted with * )

| ***APOE* signal at CHR 19** | | | | | | | | | |
| --- | --- | --- | --- | --- | --- | --- | --- | --- | --- |
| **CRP*** | **Lipids*** | | **Dementia** | | **CVD*** | **Metabolic Syndrome** | **Blood cells*** | | **Other** |
| CRP | LDL cholesterol change with statins | | Alzheimer’s disease | | Coronary artery disease | Diabetes | Platelets | | Age-related macular degeneration |
|  | APOE apolipoprotein E | |  |  |  |  |  |  |  |
|  | HDL cholesterol | | Cognitive ageing | | Chronic ischaemic heart disease | BMI/fat |  |  | Lifespan |
|  | Self reported high cholesterol | |  |  |  |  |  |  |  |
|  | Treatment with statins | | Dementia with Lewy bodies | | Myocardial infarction | Medication for cholesterol, blood pressure or diabetes: cholesterol lowering medication | Red cell distribution width | | quantity of physical activity |
|  | Treatment with cholesterol lowering medication | |  |  |  |  |  |  |  |
|  | Treatment with ezetimibe | | Posterior cortical atrophy | |  |  |  |  | pulse rate |
|  | Total cholesterol | |  |  |  |  |  |  |  |
| ***HNF1A* signal CHR 12** | | | | | | | | | |
| **CRP*** | | **Lipids*** | | **CVD*** | | **Hepatic** | | **Blood cells*** | |
|  |  | Treatment with cholesterol lowering medication | | Coronary artery disease | | Gamma glutamyl transferase | | Platelets | |
|  |  | Self-reported high cholesterol | |  |  | Cholelithiasis | |  |  |

**Supplementary Table 4**. **eQTLs for genome-wide significant SNPs from PhenoScanner database**

| **Marker Name** | **rsID** | **Tissue type** | **Gene** |
| --- | --- | --- | --- |
| chr19:45411941 | rs429358 | Lymphoblastoid cell lines | *TOMM40* |
|  |  | Whole blood | *PVRL2* |
| chr12:121423285 | rs11065384 | Blood | *OASL* |
|  |  | Blood | *C12orf43* |
|  |  | Whole blood | *CAMKK2* |
|  |  | Whole blood | *P2RX4* |
|  |  | Whole blood | *SPPL3* |
|  |  | Whole blood | *HNF1A* |
|  |  | Artery tibial | *C12orf43* |
|  |  | Thyroid | *ACADS* |

**Supplementary Table 5**. **Results of two genome-wide significant SNPs in a secondary meta-analysis of African ancestry individuals**

| **MarkerName** | **A1** | **A2** | **Freq1** | **Effect** | **StdErr** | **P value** | **N** |
| --- | --- | --- | --- | --- | --- | --- | --- |
| 19:45411941 | T | C | 0.7651 | 0.0579 | 0.0463 | 0.2116 | 1,454 |
| 12:121423285 | T | C | 0.1113 | 0.0263 | 0.0602 | 0.6615 | 1,461 |

**Supplementary Table 6.** **CRP-GWAS results for other statin response SNPs**

| **PGX Trait** | **MarkerName** | **rsID** | **Locus** | **A1** | **A2** | **Freq1** | **Effect** | **StdErr** | **P value** | **N** |
| --- | --- | --- | --- | --- | --- | --- | --- | --- | --- | --- |
| LDL | 1:109818530 | rs646776 | SORT1/CELSR2/PSRC1 | T | C | 0.76 | 0.001 | 0.010 | 0.912 | 14,070 |
| LDL | 6:161010118 | rs10455872 | LPA | A | G | 0.94 | -0.010 | 0.019 | 0.601 | 14,070 |
| LDL | 12:21368797 | rs2900478 | SLCO1B1 | A | T | 0.15 | 0.025 | 0.016 | 0.103 | 9,903 |
| HDL | 16:56989590 | rs247616 | CETP | T | C | 0.34 | -0.010 | 0.010 | 0.314 | 14,070 |
| LDL | 19:45415640 | rs445925 | APOE | A | G | 0.13 | 0.001 | 0.015 | 0.947 | 12,956 |

**Supplementary materials:**

**Statin dose equivalence Table used by the MHI-AZ cohorts:**

| Equivalent Atorvastatin | Atorvastatin | Fluvastatin | Lovastatin | Pravastatin | Rosuvastatin | Simvastatin | Cerivastatin | Itavastatine |
| --- | --- | --- | --- | --- | --- | --- | --- | --- |
| 5 mg | - | 40 mg | 20 mg | 20 mg | - | 10 mg | - | - |
| 10 mg | 10 mg | 80 mg | 40 mg | 40 mg | 5 mg | 20 mg | 0,4 mg | 2 mg |
| 20 mg | 20 mg | - | 80 mg | 80 mg | 10 mg | 40 mg | 0,8 mg | 4 mg |
| 40mg | 40 mg | - | - | - | 20 mg | 80 mg | - | - |
| 80 mg | 80 mg | - | - | - | 40 mg | - | - | - |

This is an updated version of the original statin dose equivalence based on the OHSU Drug Class Review on HMG-CoA Reductase Inhibitors, final report November 2009 (<https://www.ncbi.nlm.nih.gov/pubmedhealth/PMH0008782/pdf/PubMedHealth_PMH0008782.pdf>), also similar to what was used in our previous GIST paper (Postmus et al, Nat Comms 2014).

**Supplementary Figure 1. QC check for heterogeneity within GWAS meta-analysis**


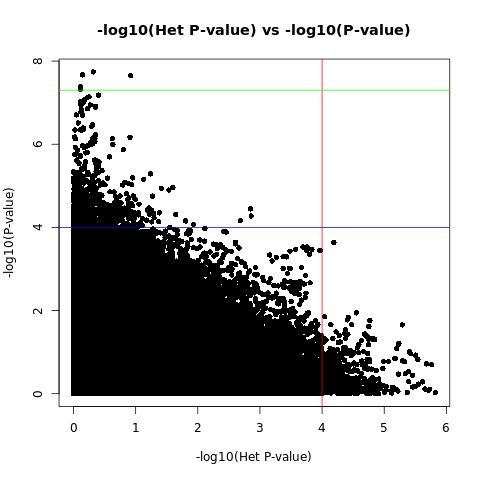

Supplement: SupplementaryDOC [file NIHMS2179082-supplement-SupplementaryDOC.docx]
